# A Pervasive Pulmonary Function Estimation System with Six-Minute Walking Test

**DOI:** 10.3390/bios12100824

**Published:** 2022-10-04

**Authors:** Ming-Feng Wu, Chi-Min Teng, Tz-Hau Kuo, Wei-Chang Huang, Chih-Yu Wen

**Affiliations:** 1 Division of Chest Medicine, Department of Internal Medicine, Taichung Veterans General Hospital, Taichung 407, Taiwan; 2 Department of Medical Laboratory Science and Biotechnology, Central Taiwan University of Science and Technology, Taichung 407, Taiwan; 3 Department of Electrical Engineering, National Chung Hsing University, Taichung 402, Taiwan; 4 Department of Post-Baccalaureate Medicine, College of Medicine, National Chung Hsing University, Taichung 402, Taiwan; 5 School of Medicine, Chung Shan Medical University, Taichung 402, Taiwan; 6 Ph.D. Program in Translational Medicine, National Chung Hsing University, Taichung 402, Taiwan; 7 Department of Medical Technology, Jen-Teh Junior College of Medicine, Nursing and Management, Miaoli 350, Taiwan; 8 Bachelor Program of Electrical Engineering and Computer Science, Innovation and Development Center of Sustainable Agriculture (IDCSA), National Chung Hsing University, Taichung 402, Taiwan

**Keywords:** six-minute walking test, forced vital capacity (FVC), forced expiratory volume in 1 second (FEV_1_), pulmonary rehabilitation, spirometry

## Abstract

Self-monitoring for spirometry is beneficial to assess the progression of lung disease and the effect of pulmonary rehabilitation. However, home spirometry fails to meet both accuracy and repeatability criteria in a satisfactory manner. The study aimed to propose a pervasive spirometry estimation system with the six-minute walking test (6MWT), where the system with information management, communication protocol, predictive algorithms, and a wrist-worn device, was developed for pulmonary function. A total of 60 subjects suffering from respiratory diseases aged from 25 to 90 were enrolled in the study. Pulmonary function test, walking steps, and physical status were measured before and after performing the 6MWT. The significant variables were extracted to predict per step distance (PSD), forced vital capacity (FVC) and forced expiratory volume in one second (FEV_1_). These predicted formulas were then implemented in a wrist-worn device of the proposed pervasive estimation system. The predicted models of PSD, and FVC, FEV_1_ with the 6MWT were created. The estimated difference for PSD was—0.7 ± 9.7 (cm). FVC and FEV_1_ before performing 6MWT were 0.2 ± 0.6 (L) and 0.1 ± 0.6 (L), respectively, and with a sensitivity (Sn) of 81.8%, a specificity (Sp) of 63.2% for obstructive lung diseases, while FVC and FEV_1_ after performing the 6MWT were 0.2 ± 0.7 (L) and 0.1 ± 0.6 (L), respectively, with an Sn of 90.9% and an Sp of 63.2% for obstructive lung diseases. Furthermore, the developed wristband prototype of the pulmonary function estimation system was demonstrated to provide effective self-estimation. The proposed system, consisting of hardware, application and algorithms was shown to provide pervasive assessment of the pulmonary function status with the 6MWT. This is a potential tool for self-estimation on FVC and FEV_1_ for those who cannot conduct home-based spirometry.

## 1. Introduction

Forced vital capacity (FVC) and forced expiratory volume in one second (FEV_1_) are the major components of the pulmonary function test with spirometry [1,2], where FVC refers to the maximum amount of air that can be exhaled when blowing out as fast as possible, and FEV_1_ is the volume of air exhaled in the first second of FVC. By means of measurement with a spirometer, the levels are often used for diagnosing the severity of pulmonary disorders and monitoring responses to treatment and intervention [1,2,3]. An existing study indicated that the ratio of FEV_1_ to FVC usually ranges from 0.70 to 0.80 in adults and is greater than 0.90 in children [4]. Although it is suggested airflow limitation as the ratio less than the criteria, an FEV_1_/FVC ratio of less than 0.70 has been widely used to define airflow limitation in adults [2]. The reversibility test with a provocation test or a bronchodilator test (BT) is conducted to confirm potential asthma, and then a post-bronchodilator test is applied to confirm chronic obstructive pulmonary disease (COPD) and its severity [5,6,7,8].

Since pulmonary rehabilitation (PR) is beneficial to the improvement of muscle force, dyspnea and health-related quality in patients with respiratory disorders (e.g., COPD, asthma, and interstitial lung disease (ILD)), spirometry and the six-minute walking test (6MWT) are often used for monitoring the effect on PR programs [9,10,11,12]. Regarding the 6MWT, this simple and submaximal exercise test provides a global and integrated response of both physical and psychological factors with the patients instructed to walk as fast as they could, where the walking distance was registered after 6 min [12,13]. Based on the guidelines of the American Thoracic Society (ATS) with a standardized approach, the 6MWT is statistically reproducible for PR [11,14].

Both spirometry and the 6MWT were instructed by well-trained medical technologists in a hospital for quality diagnosis and vital safety. However, the waiting time for the inspection is long and the cost of transportation to clinics is also high. Moreover, the hospital clusters may be reduced to deal with pandemic diseases such as coronavirus disease 2019 (COVID-19) [15]. Telemedicine PR and remote care delivery were designed to provide the connection between patients and the medical providers [16,17]. There were many successful point-of-care health services in clinical applications [18,19].

Several portable sensing devices for pulmonary function were reported in [20,21,22]. They had the features of low cost and easy use. However, they may not present all the values of FVC, FEV_1_ and FEV_1_/FVC for reference. Larson et al. [20] use a microphone on a mobile phone to diagnose varying degrees of obstructive lung ailments with a low FVC accuracy rate. Alam et al. [21] predict lung functions from recorded voice and enable patients to achieve improved symptom control. Nevertheless, it is not able to utilize a feature engineering method to identify informative features. Chun et al. [22] explore the status of lung function via a mobile phone and further predict the ratio of FEV_1_/FVC. However, the individual values of FVC and FEV are not available.

To safely provide the self-monitoring pulmonary function for progression during telemedicine or remote PR, we proposed a pervasive estimation system for the subjects of ILD or COPD. The proposed novel system consists of a measurement system using a wrist-worn device and predictive algorithms for pulmonary function, demonstrating the estimation results immediately after performing the 6MWT. By means of translational clinical data to a clinical application, the pulmonary function estimation may contribute to the feasibility of promotion with home-based PR.

This paper proposes a pervasive estimation system with telemedicine and wireless sensor networking for pulmonary function. The Section 2 depicts the proposed predicted formula and provides information of the methods and procedures used in this study. The Section 3 details the pervasive measurement and information management with a wrist-worn device. The Section 4 depicts the validation results with the characteristics of the enrolled subjects. Section 5 compares and contrasts existing works and evaluates the system’s performance. In Section 6, conclusions are presented and suggestions are made for further research.

## 2. Methods

### 2.1. Enrolled Subjects and Study Design

The subjects were enrolled if they were scheduled to undergo the 6MWT and spirometry for the assessment the impairments of respiratory diseases in Taichung Veterans General Hospital (TCVGH) between January 2015 and June 2019. A total of 60 subjects with the age from 25 to 90 were enrolled and their histories were retrospectively reviewed for the study. The records of the pulmonary function test, the BORG scale [11] and physical status before and after performing the 6MWT were also reviewed. The Institutional Review Board and Ethics Committee of TCVGH approved this study (approval number: CE19293A) and waived the need for informed consent from the participants because the study was based on a retrospective electronic medical chart review.

### 2.2. The Six-Minute Walking Test

Subjects without unstable angina or myocardial infarction (MI) and the resting heart rate, blood pressure of systolic (SBP)/diastolic (DBP) less than 120, 180/100 mm Hg, respectively, were arranged to perform the 6MWT. The conduct of the test was under the instruction of a well-trained medical technologist based on ATS guidelines [11]. Subjects were equipped with an oxygen monitor (Rossmax, SA310, Taipei, Taiwan [23]) and encouraged to walk as fast as possible around two marked cones on a path of 30 m in length for six minutes. The step counting was visually monitored by another staff member and the six-minute walking distance was recorded.

### 2.3. Pulmonary Function Test

Pulmonary function test (PFT) was conducted with a standard spirometer (Vmax Encore, Carefusion, Yorba Linda, Orange County, CA, USA). By detection from the flow sensor, a spirometer can measure the volume and flow of air when the subject inhales or exhales through the mouthpiece. Based on the standardization of the guidelines [1], the subject inspired as deeply as possible and then expired with maximal effort until there was no flow for at least 6 s for an acceptable FVC trial. The acceptable maneuvers were repeated three times. Once both differences between the largest and the next largest FVC and FEV_1_ < 0.150 L were met, the pulmonary function test was determined with the trial of the largest value of FVC plus FEV_1_. The predicted values of FVC (defined as FVCpred) and FEV_1_ (defined as FEV_1_pred) were adapted from [24].

### 2.4. BORG Scale

The BORG scale was an assessment tool with simple numerical list for dyspnea and overall fatigue [11,25]. The set of integers ranging from 0 to 10, respectively, represented degrees of the scale from nothing at all to very severe. Subjects were asked to rate their exertion on the scale before and after 6MWT.

### 2.5. The Predicted Formula Development

To develop the predicted formulas and test the validation for pulmonary functions, enrolled subjects were stratified for sampling based on body height and categorized into two sets. One set was for training and the rest set for validation. Set comparisons were conducted with the independent t-test for continuous variables and the chi-square test for categorical variables (Table 1). Physiological factors with Pearson correlation > 0.4 for FVC, FEV_1_, per step distance (PSD), respectively, were the independent variable for significant feature extraction of stepwise regression on a training set to develop predicted models [26].

Regarding the training set, the baseline pulmonary functions of FVC, FEV_1_, FEV_1_/FVC were 2.3 ± 0.60 (L), 1.6 ± 0.6 (L) and 70.5 ± 22.3 (%), respectively, and resting SpO_2_ was 95.5 ± 2.3 (%). When performing the 6MWT, the walking procedure was recorded by counting total steps (TS) of 634.4 ± 127.2 with the total distance (TD) of 416.1 ± 93.7 (m). Furthermore, the SpO_2_nidar (%) as well as peak of heart beat were 85.5 ± 5.7 (%) and 140.1 ± 32.1, respectively, during the 6MWT, while the Borg scale of the end point of 6MWT was 4.4 ± 1.4. There was no significant difference in those variables between the training and validation datasets.

Based on the TS and TD measurements, the per step distance (PSD) was calculated with the ratio of the TD to the TS. There were three significant correlated variables for PSD (Table 2). Stepwise regression showed that with R^2^adj = 0.339, PSD was estimated with

(1) $${\mathrm{PSD}}_{\mathrm{estd}}\left(m\right)=0.289+0.153\times \;{\mathrm{FEV}}_{1}\mathrm{pred}\;\left(L\right)$$

Both FVC and FEV_1_ before the 6MWT were with five significant correlated variables (i.e., FVC, FEV_1_, per step distance (PSD), FEV_1_/FVC and FVC/FVCpred). With R^2^adj = 0.476, the estimated FVCpre was trained with:

(2) $${\mathrm{FVCpre}}_{\mathrm{estd}}\left(L\right)=-4.625+0.035\times \mathrm{body}\;\mathrm{height}\left(\mathrm{cm}\right)+1.810\times \mathrm{PSD}\left(\mathrm{cm}\right)\;$$

and with R^2^adj = 0.356, estimated FEV_1_pre was trained with:

(3) $${\mathrm{FEV}}_{1}\mathrm{pre}\_\mathrm{estd}\;\left(L\right)=-0.328+0.787\times {\mathrm{FEV}}_{1}\mathrm{pred}\;\left(L\right)$$

Note that once PSD was determined, FVCpre may be calculated accordingly. Besides, PSD may be estimated with Formula (2) for the unknown TD or TS. FVC and FEV_1_ after 6MWT were also with the above five significant correlated variables. With R^2^adj = 0.470 and 0.317, respectively, the estimated FVCpost and FEV_1_post were trained with:

(4) $${\mathrm{FVCpost}}_{\mathrm{estd}}\left(L\right)=-4.249+0.031\times \mathrm{body}\;\mathrm{height}\;\left(\mathrm{cm}\right)+2.255\times \;\mathrm{PSD}\left(\mathrm{cm}\right)$$

and

(5) $${\mathrm{FEV}}_{1}\mathrm{post}\_\mathrm{estd}\;\left(L\right)=-0.235+0.752\times \;{\mathrm{FEV}}_{1}\mathrm{pred}\;\left(L\right)$$

### 2.6. Statistics

To verify the accuracy of predicted formulas, the comparisons were made for the subjects on the validation set. A previous study of prediction for PSD with body height (in centimeters) minus 100, defined as PSD_H100 was used for the comparison [27]. The Bland–Altman plot was used to compare the predictions and actual values for FVC, FEV_1_ as well as the PSD. In addition, sensitivity and specificity were used to assess the airway obstruction based on FEV_1_/FVC < 70% before (pre-exercise) and after (post-exercise) performing the 6MWT as well as FEV_1_/FEV_1_pred. Note that all variable values were presented as mean ± standard deviation (sd) for continuous variables and as frequencies (%) for categorical variables. Statistical analysis was performed using the SPSS version 18.0 (SPSS, Chicago, IL, USA) and *p*-value < 0.05 was set for the significance.

## 3. Pervasive Estimation System

Once the predicted formulas were confirmed, they were embedded in the pervasive measurement system. The input values were obtained from the sensing of the system hardware and the physiological variables of the subjects. The transmission protocol and user interface were established for the system prototype. Besides, the feasibility of pulmonary function prediction for the measurement system with the 6MWT was also evaluated in the study. This section details the system architecture, including information management, communication protocol, and hardware/software implementation.

### 3.1. Information Management

Due to the difficulties of integrating the clinical data from different operational data sources (e.g., symptoms, physical examinations, and laboratory test results), back-end management systems were developed to handle these scenarios. The information management consisted of two major components: (1) a database server MySQL and (2) a management tool phpMyAdmin for increasing system flexibility. In this work, with Apache and PHP web server modules, an information management system was developed for the 6MWT assessment. For recording the 6MWT assessment data, a MySQL database can be implemented as a single application in a client/server network environment, or as a database added to other software, and it also has a major function of managing, checking, and optimizing the management tools of the database. Accordingly, the MySQL sends instructions to the assigned database server, and then the server returns the execution result to MySQL to form a loop.

### 3.2. Communication Protocol

**Figure 1:**
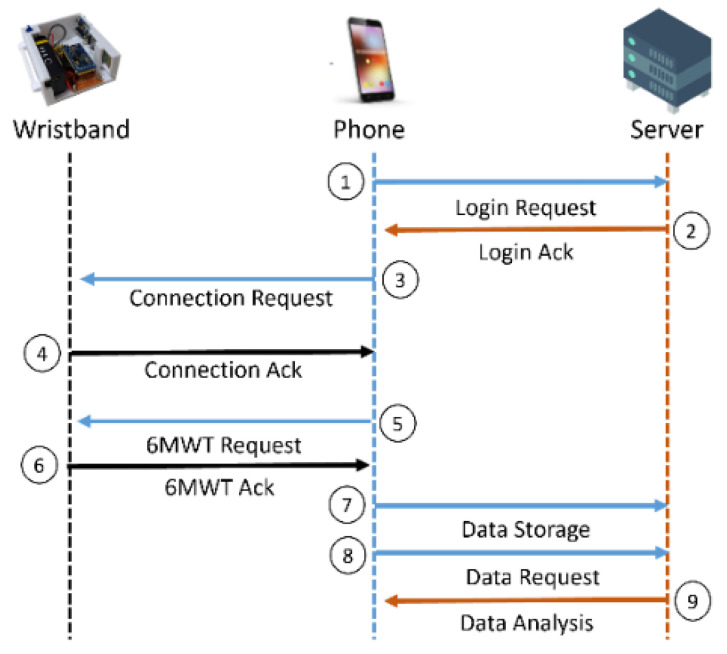
Communication protocol.

Figure 1 presents the communication protocol of the proposed system. In Steps 1 and 2, the phone is used to make a login request to the server and get a response from the server. In Steps 3 and 4, the phone initiates a communication to a wristband device and then establishes a communication channel. In Step 5, the phone selects the 6MWT operation mode and sends exercise instructions to the wristband device. In Step 6, after completing the assessment, the wristband device sends the 6MWT data to the phone. In Step 7, the phone forwards the recorded data to the server for storage. In Steps 8 and 9, the phone may initiate a request to the server for historical observations and data analysis.

Based on the communication protocol, the Arduino mini firstly collects the PPG signal and the angular rate via an optical sensor and a three-axis accelerometer gyro sensor. Then, a smart phone communicates with the Arduino mini via the Bluetooth Low Energy (BLE) wireless technology. Finally, the Internet is applied to establish a channel between a smart phone and a medical nursing center.

### 3.3. Hardware/Software Implementation

**Figure 2:**
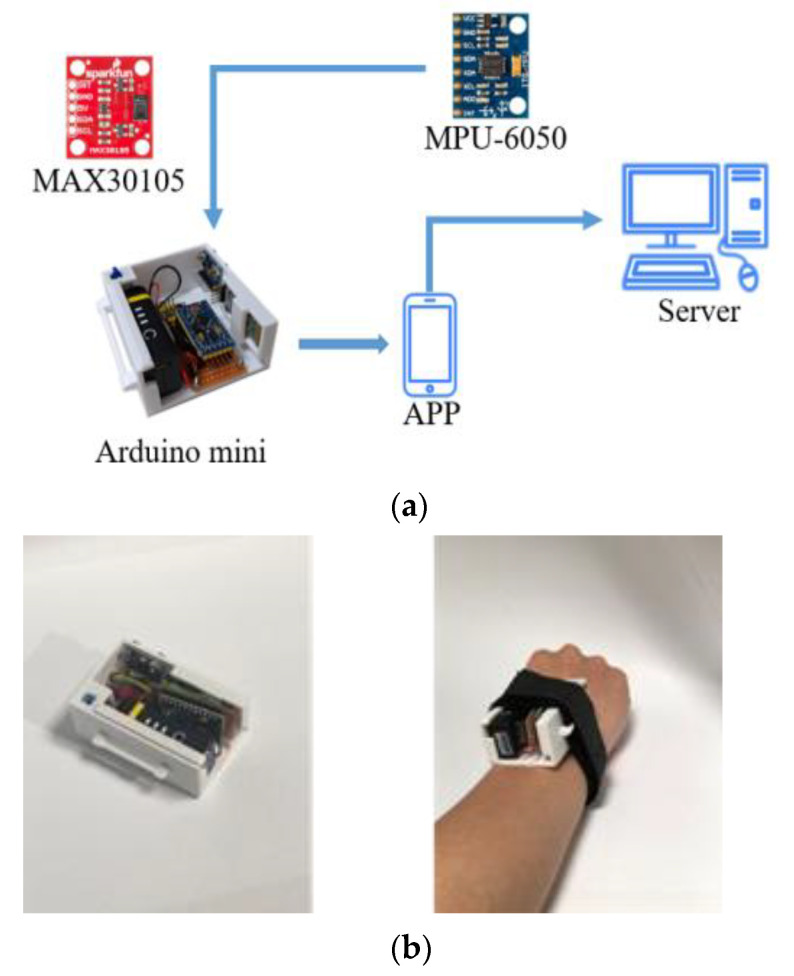
The components and the prototype of the system. (**a**) System implementation; (**b**) a wristband prototype.

As shown in Figure 2a, the MPU6050 was used for measuring human walking speed through red, green and IR LED. The SparkFun MAX30105 particle sensor was used for monitoring of heart rate. These sensors and components were integrated with a small device strapped to the patient’s wrist (Figure 2b). Arduino delivered the processed date to a smart phone with Bluetooth. A smart phone saved the sensing data to the SQLite, and then uploaded the 6MWT information to the database through the Internet. On the patient side, their 6MWT history can be displayed on a smart phone. On the hospital side, the patient’s use record can be illustrated on the website.

With the communication protocol as described in Figure 1, the wristband device estimates the user’s walking pace, time, and distance. At the end of the exercise, the data is forwarded to the database, and then users can query their own 6MWT records through the application (APP). Moreover, to validate the accuracy of counting walking steps with the prototype device as depicted in Figure 2, two researchers of the study volunteered to perform the 6MWT exercise with the fast and slow walking speeds around two marker cones, 30 m apart, 10 times.

## 4. Results

**Figure 3:**
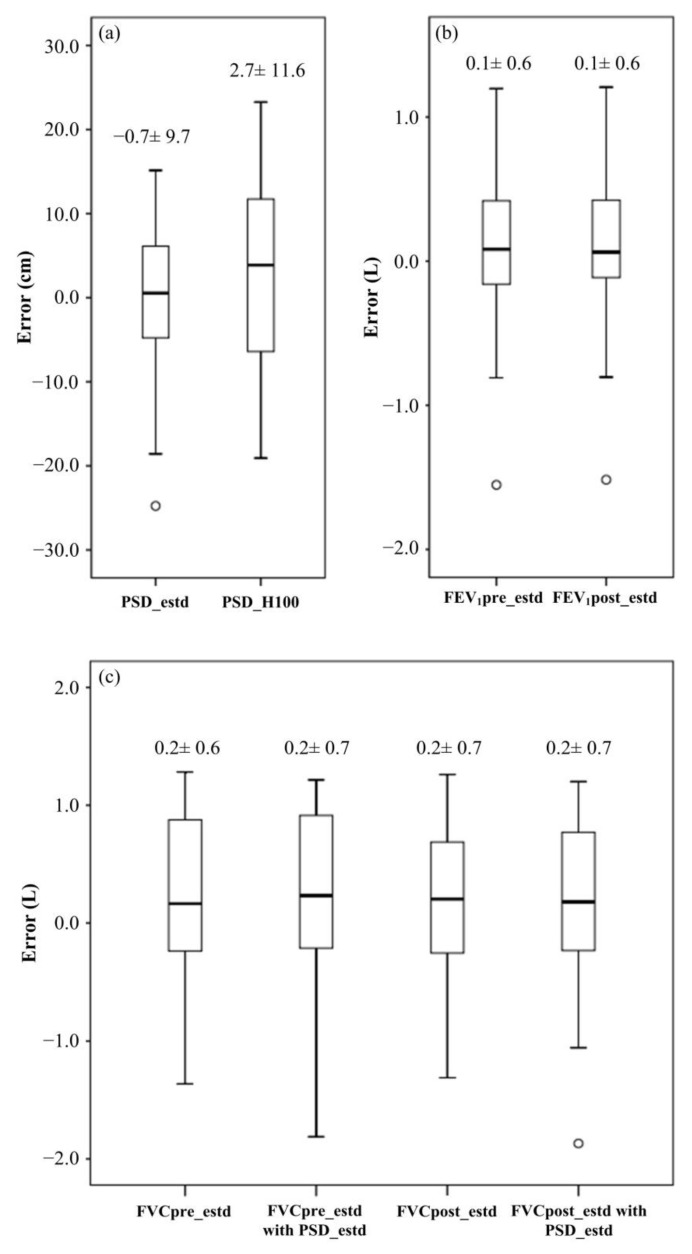
The box plot of predicted errors for the validation set. (**a**) Per step distance; (**b**) FEV_1_; (**c**) FVC. Note that the circle symbols represent the outliers.

Half of the 60 participants were with chronic obstructive disease (COPD) or interstitial lung disease (ILD) in the study (Table 1). With the five estimated models (Formulas (1)–(5)), the PSD, FVC and FEV_1_ were validated. The study showed that the predicted errors of −0.7 ± 9.7(cm) for PSD with PSD_estd were smaller than that of PSD_100 (Figure 3 and Table S1). Moreover, as shown in Table S1, FEV_1_pre_estd and FVCpre_estd were with predicted errors of 0.1 ± 0.6 (L) and 0.2 ± 0.6 (L), respectively, while the predicted errors of FEV_1_pre_estd/FVCpre_estd (%) and FEV_1_post_estd/FVCpost _estd (%) were −1.0 ± 17.4 and −1.0 ± 17.9, respectively. Since PSD was determined by the ratio of TD to TS, there would be no PSD value as performing 6MWT outdoors with the TD of walking. The result showed that using PSD_estd instead of PSD had equal performance on estimating FVCpre. The proposed estimated models were with small predicted errors for FVCpost and FEV_1_post. Furthermore, using PSD_estd to estimate FVCpost also presented equal performance with respect to PSD.

Referring to the criterion FEV_1_/FVC < 70%, which is an important indicator for airway obstruction, the study calculated the estimated values for the prediction and showed that sensitivity, specificity and accuracy before 6MWT (pre-exercise) were 81.8, 63.2 and 70.0%, respectively (Table 3). For the post-exercise, sensitivity, specificity and accuracy were 90.9, 63.2 and 73.3%, respectively. The differences in FVC and FEV_1_ between pre-exercise and post-exercise were 0.02 ± 0.11 (L) and −0.02 ± 0.07 (L), respectively.

**Figure 4:**
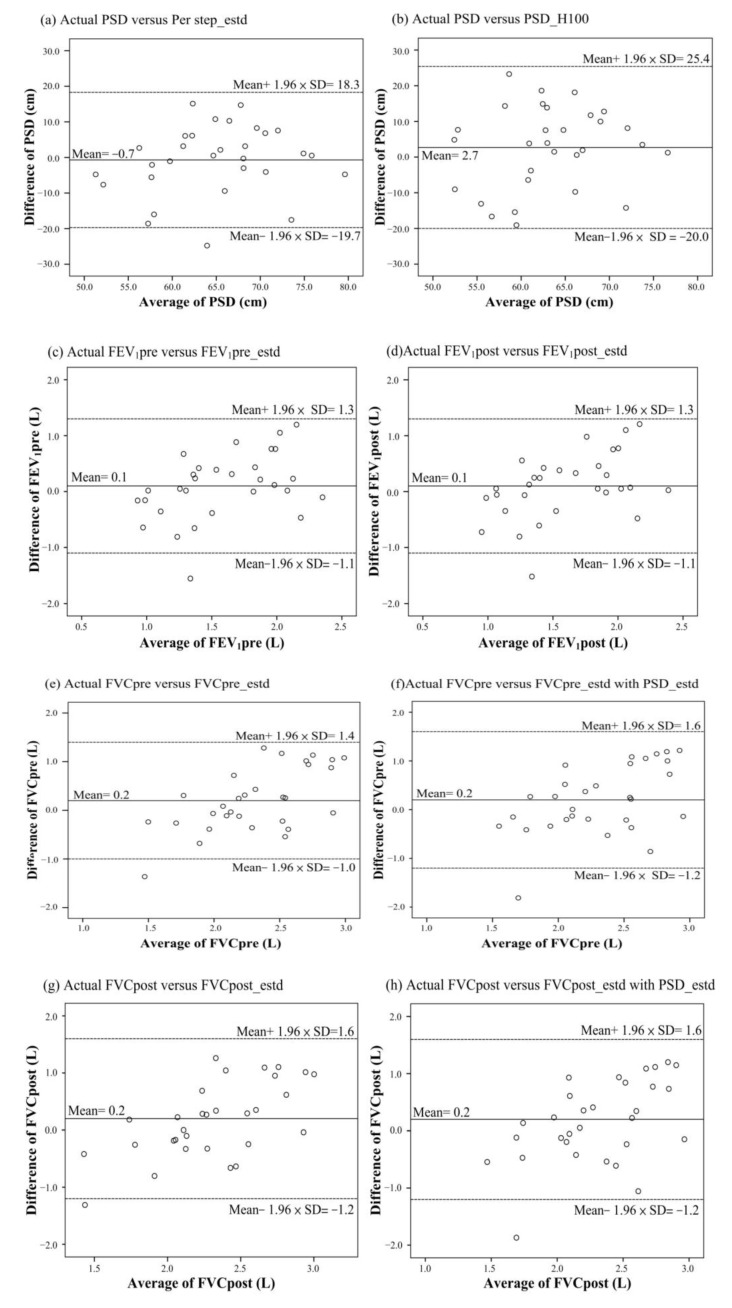
Bland–Altman plot between predictions and actual value. (**a**) Actual PSD versus PSD_estd; (**b**) Actual PSD versus PSD_H100; (**c**) Actual FEV_1_pre versus FEV_1_pre_estd; (**d**)Actual FEV_1_post versus FEV_1_post_estd; (**e**) Actual FVCpre versus FVCpre_estd; (**f**) Actual FVCpre versus FVCpre_estd with PSD_estd; (**g**) Actual FVCpost versus FVCpost_estd; (**h**) Actual FVCpost versus FVCpost_estd with PSD_estd.

Bland–Altman plot showed that PSD_estd presented a better agreement to the actual PSD with a difference of −0.7 cm compared with previous studies in [27] (Figure 4a,b). Observe that the predicted difference of FVCpre_estd and FVCpre_estd with PDS_estd was the same with a mean of 0.2 L (Figure 4e,f). Moreover, there was an equal difference between the pre-exercise and post-exercise for FVC (Figure 4c–e,g) for FEV_1_. However, there was an estimated value outside the 95% confidence interval (CI) at [mean of difference −1.96 × SD] for PSD, FVC and FEV_1_ based on the proposed estimated Formulas (1)–(5).

**Figure 5:**
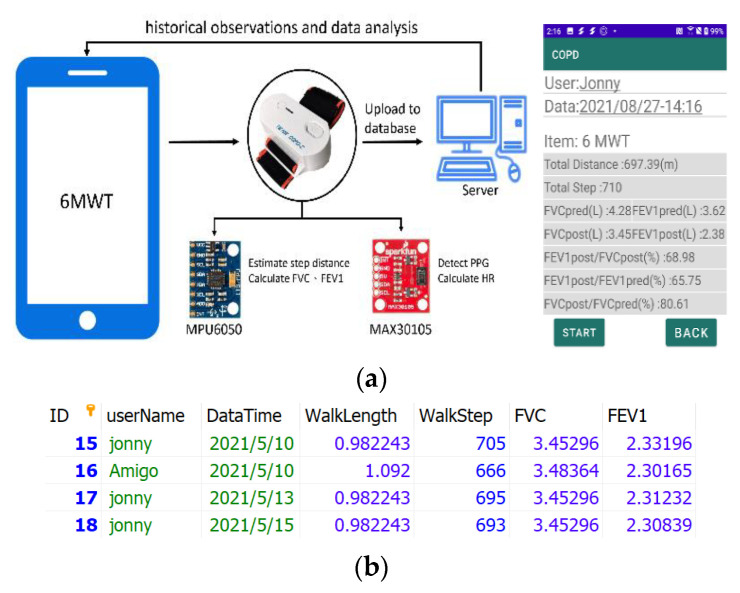
(**a**) The process chart (left) and home screen (right); (**b**) the 6MWT history (SQL).

After evaluating the proposed estimation models, the performance of the proposed information management system and the accuracy of counting walking steps were examined. Two researchers with the age and body height (cm) of 47, 168 and 23, 182, respectively, participated in the measurement of step counting with 580.5 ± 101.5 for the pervasive estimation system. Given the real steps were 595.5 ± 111.9. The counting difference was −1.6 ± 15.4. Furthermore, in Figure 5, with the 6MWT assessment, the data was transferred from the wristband to the phone. Then, the mobile phone calculated FVC and FEV_1_ based on the user’s gender, height, age, weight and the walking distance of six minutes. For the purpose of clinical diagnosis, the recorded data is sent to SQL, and the doctor can obtain the patient’s 6MWT data from the database to further assess the patient’s pulmonary function.

## 5. Discussion

The study proposed five estimated models for PSD, FVCpre, FEV_1_pre, FVCpost and FEV_1_post with a statistic power of 92.2, 99.6, 94.1, 99.5 and 89.4%, respectively. Based on these models, the results showed that the sensitivity (Sn) and specificity (Sp) of obstructive lung diseases were 81.8 and 63.2% for pre-exercise and 90.9 and 63.2% for post-exercise. These formulas were embedded in the pervasive measurement system with a wristband prototype. With basic variables (e.g., age, sex), the proposed system can display the pulmonary function with the 6MWT. The prototype of the pervasive estimation system showed that the step counting was more accurate with the difference of −1.6 ± 15.4 than the previous study of −3.83 ± 22.05 with the Sportline brand and another three commercial pedometers [28]. Previous work reported that the walking distance in the 6MWT was associated with spirometry [29,30]. A total of steps in the 6MWT was also efficient in evaluating the functional status of COPD patients [31]. The results supported the proposed pervasive measurement system with the 6MWT for the estimation of pulmonary function.

A home-based mobile spirometer was a tool for self-monitoring in daily pulmonary function at home for Duchenne Muscular Dystrophy (DMD) and the detecting progression in idiopathic pulmonary fibrosis (IPF). However, it took a while for the training to use the device [32,33]. A home-based mobile spirometer was also used to reduce the frequency of acute exacerbation of COPD [34]. Piotr et al. reported 10,936 spirometries in 9855 patients were performed by 673 primary care professionals with a mobile phone-linked portable device, showed 5347 (49%) spirometry examinations met both accuracy and repeatability criteria [32]. There may be more performance plateau errors for self-using in the general population. The 6MWT is a simple and reproducible measurement [11,12,13,14]. The proposed system estimated pulmonary function with the 6MWT, which may decrease the bias without maximal effort or exhalation until no flow for at least 6 s when self-performing spirometry with a home-based mobile device [35].

Many other portable sensing devices for pulmonary function were reported in [20,21,22]. They had the features of low cost and easy use. However, as shown in Table 4, they may not present all the values of FVC, FEV_1_ and FEV_1_/FVC for reference. The proposed system used body weight and PSD, and modified FEV_1_pred [24] to estimate FVC and FEV_1_, respectively. The reported FEV_1_/FVC can be used to screen obstruction with an accuracy of 70.0 and 73.3% for pre-exercise and post-exercise, respectively. Even when the total walking distance was not known outdoors, the system can still estimate the total distance through PSD prediction.

The strengths of this study include the fact that subjects with several common chronic respiratory diseases were all enrolled in the analysis, making our results more generalizable. This compensates for the inherent limitation of our study that patients with heart diseases and those who toddled or were unable to walk may not perform 6MWT, making it impossible to apply the proposed system. However, there was a case with an estimated value outside the 95% CI for a large predicted error in PSD, FEV_1_ and FVC plotted in Figure 4a,c–h of the study. When reviewing the history record, the case was with an FEV_1_ of 0.56 (L) and FVC of 0.79 (L). The low pulmonary function restricted the patient to total walking steps of 351 and distances of 181 m. Once the proposed pervasive measurement system was put into practice, the low values of walking counting will be cautionary. Nevertheless, the proposed system of the study gives the potential to conduct the clinical trial in the next work.

## 6. Conclusions

We have proposed a pulmonary function estimation system with a wrist-worn device. The wearable system can pervasively estimate the pulmonary function through the six-minute walking test. The system can be used as a substitute for spirometry to monitor the status of lung function during home-based PR. Furthermore, it may also estimate FVC and FEV_1_ with the subject failing to perform acceptable spirometry.

## Figures and Tables

**Table 1:** The characteristics of the enrolled subjects (*n* = 60).

	All (*n* = 60)	Training (*n* = 30)	Validation (*n* = 30)	*p*-Value
Age (years old)	63.4 ± 12.5	62.6 ± 11.7	64.3 ± 13.3	0.601
Body height (cm)	161.7 ± 7.8	161.6 ± 8.1	161.8 ± 7.7	0.909
Body weight (Kg)	60.1 ± 8.6	58.9 ± 10.2	61.4 ± 6.7	0.275
BMI (Kg/m^2^)	23.0 ± 3.1	22.5 ± 2.9	23.6 ± 3.2	0.170
FVCpre (L)	2.4 ± 0.63	2.3 ± 0.6	2.4 ± 0.7	0.473
FEV_1_pre (L)	1.6 ± 0.6	1.6 ± 0.6	1.6 ± 0.6	0.723
FVCpost (L)	2.34 ± 0.64	2.3 ± 0.6	2.4 ± 0.7	0.536
FEV_1_post (L)	1.62 ± 0.6	1.6 ± 0.6	1.7 ± 0.6	0.678
FEV_1_pre/FVCpre (%)	69.6 ± 20.8	70.5 ± 22.3	68.6 ± 19.6	0.727
FEV_1_post/FVCpost(%)	70.7 ± 20.9	71.3 ± 22.3	70.2 ± 19.8	0.839
FEV_1_pre/FEV_1_pred (%)	67.3 ± 22.0	64.8 ± 20.1	70.0 ± 23.8	0.400
FEV_1_post/FEV_1_pred (%)	68.0 ± 22.3	65.4 ± 20.7	70.6 ± 23.9	0.369
FVCpre/FVCpred (%)	80.0 ± 20.2	76.7 ± 16.7	82.4 ± 23.1	0.278
FVCpost/FVCpred (%)	79.3 ± 21.4	76.7 ± 18.5	82.0 ± 23.9	0.342
Peak of heart beat	136.3 ± 25.7	140.1 ± 32.1	132.6 ± 16.8	0.261
SpO_2_pre (%)	95.3 ± 2.3	95.5 ± 2.3	95.1 ± 2.3	0.503
SpO_2_nidar (%)	84.7 ± 5.9	85.5 ± 5.7	83.9 ± 6.0	0.285
Total distance (m)	418.1 ± 99.9	420.1 ± 107.3	416.1 ± 93.7	0.879
Total steps	638.9 ± 116.5	634.4 ± 127.2	643.3 ± 106.6	0.772
Per step distance (m)	0.7 ± 0.10	0.7 ± 0.1	0.6 ± 0.1	0.589
Borg scale	4.4 ± 1.3	4.4 ± 1.4	4.3 ± 1.2	0.845
COPD	33 (55.0)	17 (56.7)	16 (53.3)	0.500
ILD	33 (55.0)	17 (56.7)	16 (53.3)	0.500
Asthma	8 (13.3)	3 (10.0)	5 (16.7)	0.353
Bronchiectasis	6 (10.0)	2 (6.7)	4 (13.3)	0.335

COPD: chronic obstructive pulmonary disease; ILD: interstitial lung disease.

**Table 2:** Predicted models for dependent variables.

Estimation	Significant Factor	Correlation Coefficient	Stepwise Regression Model	Statistical Power (%)
PSD (m)	FVCpred	0.592	Formula (1) (R^2^adj = 0.339)	92.2
	FEV_1_pred	0.602		
	Body height	0.488		
FVCpre (L)	FVCpred	0.586	Formula (2) (R^2^adj = 0.476)	99.6
	FEV_1_pred	0.551		
	PSD	0.584		
	Body height	0.646		
	Body weight	0.474		
FEV_1_pre (L)	FVCpred	0.597	Formula (3) (R^2^adj = 0.356)	94.1
	FEV_1_pred	0.615		
	PSD	0.418		
	TD	0.457		
	Body height	0.408		
FVCpost (L)	FVCpred	0.539	Formula (4) (R^2^adj = 0.470)	99.5
	FEV_1_pred	0.500		
	PSD	0.619		
	Body height	0.608		
	Body weight	0.465		
FEV_1_post (L)	FVCpred	0.575	Formula (5) (R^2^adj = 0.317)	89.4
	FEV_1_pred	0.584		
	PSD	0.410		
	TD	0.413		
	Body height	0.408		

PSD: per step distance; TD: total distance.

**Table 3:** The predicted accuracy for obstruction (FEV_1_/FVC < 70%) in the validation set (*n* = 30) with the proposed estimated models.

	Pre-Exercise (Obstruction, *n* = 11)	Post-Exercise (Obstruction, *n* = 11)
True positive (*n*)	9	10
False positive (*n*)	7	7
False negative (*n*)	2	1
True negative (*n*)	12	12
Sensitivity (%)	81.8	90.9
Specificity (%)	63.2	63.2
Accuracy (%)	70.0	73.3

**Table 4:** Comparison of portable devices for lung function.

Systems	Features	Limitations	Performance
Larson [20]	Able to diagnose varying degrees of obstructive lung ailments. Use a microphone on a mobile phone.	In the measurement process, there is a sound 1 s before the exhalation, but the following sound cannot be detected. The FVC accuracy rate is low.	Mean error of 5.1% for LF.
Alam [21]	Enable patients to achieve improved symptom control. Availing of early and appropriate medication and reducing costs. Predict lung functions from recorded voice.	Unable to utilize a feature engineering method to identify informative features. The use of Pearson correlation coefficient calculated the correlation between the features and FEV_1_%.	Accuracy of 73.2% for severity of LF. Accuracy of 85.0% for predicting abnormal LF.
Chun [22]	Predict the ratio of FEV_1_/FVC. Understand the status of lung function via a mobile phone.	Unable to find out the individual values of FVC and FEV.	Accuracy of 73.7% for pathological class. Error of 8.6% for FEV_1_/FVC.
Proposed system	Perform 6MWT without a mobile phone. Subjects with common chronic respiratory diseases were enrolled in the analysis, making our results more generalizable.	Patients with heart diseases and those who toddled or were unable to walk may not perform 6MWT.	Error of 0.2 for FVC, 0.1 for FEV_1_. Sn and Sp were 90.9% and 63.2%, respectively, for obstruction LF (post-exercise). Error of −1.0 (%) for FEV_1_/FVC

LF: lung function; FEV: forced expiratory volume; FVC: forced vital capacity; 6MWT: six-minute walking test; Sn: sensitivity; Sp: specificity.

## Data Availability

Not applicable.

## Supplementary Materials

The following supporting information can be downloaded at: https://www.mdpi.com/article/10.3390/bios12100824/s1, Table S1: The predicted errors for the validation set.
