# Supplementary material for: A Pervasive Pulmonary Function Estimation System with Six-Minute Walking Test
Source: Biosensors (Basel). 2022 Oct 4;12(10):824. doi: 10.3390/bios12100824 (PMC9599376; doi:10.3390/bios12100824)
Supplement: Supplementary file 1 [file biosensors-12-00824-s001.zip › biosensors-1889175-supplementary.pdf]

Supplementary Table S1. The predicted errors for the validation set.

Item	Estimated model	Difference
PSD (cm)	PSD_estd	-0.7 ± 9.7
	PSD_H100	2.7 ± 11.6
FEV_1pre (L)	FEV_1pre_estd	0.1 ± 0.6
FEV_1post (L)	FEV_1post_estd	0.1 ± 0.6
FVCpre (L)	FVCpre_estd	0.2 ± 0.6
	FVCpre_estd with PSD_estd	0.2 ± 0.7
FVCpost (L)	FVCpost_estd	0.2 ± 0.7
	FVCpost_estd with PSD_estd	0.2 ± 0.7
FEV_1pre/FVCpre (%)	FEV_1pre_estd/ FVCpre_estd	-1.0 ± 17.4
FEV_1post/FVCpost (%)	FEV_1post_estd/ FVC post _estd	-1.0 ± 17.9
FEV_1pre/ FEV_1pred (%)	FEV_1pre_estd /FEV_1pred	5.4 ± 24.4
FEV_1post/ FEV_1pred (%)	FEV_1post_estd / FEV_1pred	5.7 ± 24.3
